# Nutritional Supplements Fortified with Oils from Canola, Flaxseed, Safflower and Rice Bran Improve Feedlot Performance and Carcass Characteristics of Australian Prime Lambs

**DOI:** 10.3390/ani8120231

**Published:** 2018-12-05

**Authors:** Hung V. Le, Quang V. Nguyen, Don V. Nguyen, Bunmi S. Malau-Aduli, Peter D. Nichols, Aduli E. O. Malau-Aduli

**Affiliations:** 1 Animal Genetics and Nutrition, Veterinary Sciences Discipline, College of Public Health, Medical and Veterinary Sciences, Division of Tropical Health and Medicine, James Cook University, Townsville, QLD 4811, Australia; vanhung.le@my.jcu.edu.au (H.V.L.); quang.nguyen2@my.jcu.edu.au (Q.V.N.); donviet.nguyen@my.jcu.edu.au (D.V.N.); Peter.Nichols@csiro.au (P.D.N.); 2 National Institute of Animal Science, Thuy Phuong, Bac Tu Liem, Hanoi 129909, Vietnam; 3 College of Economics and Techniques, Thai Nguyen University, Thai Nguyen 252166, Vietnam; 4 College of Medicine and Dentistry, Division of Tropical Health and Medicine, James Cook University, Townsville, QLD 4811, Australia; bunmi.malauaduli@jcu.edu.au; 5 CSIRO Oceans & Atmosphere, P.O. Box 1538, Hobart, TAS 7001, Australia

**Keywords:** PUFA, oil, prime lamb, feedlot, carcass characteristics, live performance, oils, canola, flaxseed, safflower, rice bran

## Abstract

**Simple Summary:**

This study evaluated the feedlot response of Australian prime lambs to supplementation with oil based polyunsaturated fatty acid enriched pellets. The results demonstrated that live animal performance and carcass characteristics of prime lambs on a lucerne basal diet were improved after the supplementation with oil based polyunsaturated fatty acid enriched pellets. Supplementation of lambs with rice bran oil and canola oil resulted in improved live animal performance and carcass characteristics of prime lambs at comparatively lower feed costs than oils from flaxseed, safflower and rumen-protected sources. These results are very useful for prime lamb producers in increasing product quality and farm profitability without compromising animal performance and well-being.

**Abstract:**

This study investigated live animal performance and carcass characteristics of Australian prime lambs fed oil based polyunsaturated fatty acid (PUFA) enriched pellets in a feedlot system. The tested hypothesis was that supplementation of lambs with a variety of dietary oil based PUFA enriched pellets would enhance growth and carcass characteristics compared with the control lambs fed only with lucerne hay. Seventy-two, 6 months old White Suffolk x Corriedale first-cross prime lambs with an average liveweight (LWT) of 35.7 ± 0.9 kg were allocated to six treatment groups in a completely randomised experimental design. The treatments were: (1) control: lucerne hay only; or lucerne hay plus wheat-based pellets infused with 50 mL/kg dry matter (DM) of oils from (2) rice bran (RBO); (3) canola (CO); (4) rumen protected (RPO); (5) flaxseed (FO) and (6) safflower (SO) dietary sources. All lambs had *ad libitum* access to lucerne hay and clean fresh water. Supplemented lambs were fed 1 kg of pellet/head/day for 10 weeks. Feed intake, final LWT, average daily gain (ADG), body conformation and carcass characteristics of lambs in the supplemented groups were all greater than for the control group. SO lambs had the lowest ADG of 190.3 g/day. RBO and CO treatments had the lowest feed cost per unit gain of AU$3.0/kg. Supplemented lambs had similar over the hooks (OTH) incomes that were all higher than that of the control group. This empirical evidence-based data demonstrated that supplementation of lambs with RBO and CO had comparatively lower feed costs without compromising ADG, carcass characteristics and OTH income.

## 1. Introduction

The Australian sheep industry has undergone significant changes within the last decade and witnessed a sustained decrease in the value and scale of wool and a steady rise in production and price of lamb and mutton [1]. Australia was the second largest producer of lamb and mutton in the world from 2010 to 2016 [2] and maintenance of the global competitiveness of Australian meat production ensured the sustainable development of its lamb industry. Pethick et al. [3] revealed that meat with healthy nutritional composition is one of the five key attributes of modern meat products in a competitive market. 

There are new emerging demands from meat consumers for high quality lamb meat, especially in developed countries [4,5]. Red meat contains natural omega-3 long-chain (≥ C20) polyunsaturated fatty acids (n-3 LC-PUFA), the content of which can be manipulated by modifying the composition of livestock feeds [6]. n-3 LC-PUFA are well known for human health benefits including anti-inflammatory, therapeutic and protective effects against cardiovascular diseases and various types of cancer [7,8,9,10]. However, it is challenging to increase n-3 LC-PUFA content in red meat because of lipolysis and extensive biohydrogenation that occurs in the rumen through microbial activity in ruminants [11,12]. Furthermore, in some instances, adding oil based PUFA supplements to ruminant rations resulted in reduced animal feed intake, animal performance and carcass muscle mass [13,14] or a depression of ruminal fermentation [15]. High feed cost is another challenge in the on-farm application of oil based n-3 LC-PUFA supplementation because the cost of nutrition can represent approximately 70% of the total cost of lamb production in confined systems [16,17].

Supplementation of lamb diets with n-3 LC-PUFA has shown several positive results [18,19,20,21,22,23,24,25,26,27]. The impact of fatty acids has been reported in a separate stand-alone manuscript (currently under review) where these oil supplements were shown to improve the health-beneficial n-3 LC-PUFA contents in the muscle, liver, heart and kidney of these supplemented prime lambs. However, there is still the need to examine other cost-effective and nutritionally viable oil-based PUFA dietary sources for quality prime lamb production. To our current knowledge, available published information is generally scarce on the impact of different supplemental oil based PUFA dietary sources on animal performance and associated feed costs in the Australian prime lamb feedlot industry. Therefore, the present study aims to determine the responses of Australian prime lambs, in terms of live animal performance, carcass characteristics as well as feed costs, to a variety of dietary supplemental oils from canola, rice bran, flaxseed, safflower and rumen-protected sources.

## 2. Materials and Methods

### 2.1. Animal Ethics

This study was carried out at the Tasmanian Institute of Agriculture’s Cressy Research and Demonstration Station, Burlington Road, Cressy, Tasmania, Australia from April to June 2016. The use of animals and procedures performed in this study were all approved by the University of Tasmania Animal Ethics Committee (Permit No. A0015657).

### 2.2. Animals, Diets and Experimental Design

**Figure 1:**
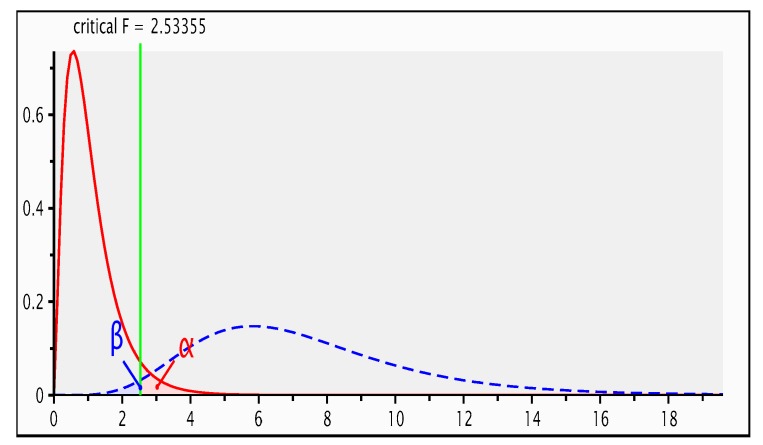
G-Power analysis for statistical power, effect and sample size.

An a priori power analysis with repeated measures was conducted using G-Power to justify an appropriate sample and effect size. As depicted in Figure 1, a minimum total sample size of 36 lambs was sufficient for a large effect size, statistical power of 95% and two-sided significance level of 0.05 in an experimental design utilising 6 treatment groups and 7 repeated measurements. Therefore, the use of 72 animals in 6 treatment groups was a statistically robust experimental design.

Seventy-two (White Suffolk x Corriedale first-cross) prime lambs with an average LWT of 35.7 ± 0.9 kg weaned at 6 months were randomly allocated into six treatments: (1) Control: lucerne hay only; or lucerne hay plus wheat-based pellet infused with 50 mL/kg DM of oil from (2) rice bran (RBO); (3) canola (CO); (4) rumen-protected (RPO), (5) flaxseed (FO) and (6) safflower (SO) sources in a completely randomised design balanced by equal number of ewe and wether lambs. The animals were kept in individual pens and had *ad libitum* access to clean fresh water and lucerne hay throughout the feeding trial. Each lamb in the supplemented treatments was fed 1 kg pellets/day. The feeding trial lasted 10 weeks including a 3-week adaptation period followed by 7 weeks of full supplementation. The freshly weighed feed was offered every day at 9:00 h after the collection and weighing of the previous day’s feed residues. Details of the experimental lamb management techniques and data collection had been extensively described in previous experiments [20,21,24,25,26,27].

### 2.3. Feed Intake, Body Conformation and Liveweight Measurements

Lucerne hay and pellet intakes were separately calculated using the difference between daily amount of total feed offered and the residual feed measured. Feed conversion efficiency and feed cost per liveweight gain were computed as described in detail by Flakemore et al. [19]. Lucerne hay cost was calculated based on the market price during the experimental period (AU$0.575/kg) and concentrate price was based on the market ingredient costs of AU$0.160/kg, AU$0.194/kg, AU$0.534/kg, AU$0.486/kg, AU$0.778/kg for RBO, CO, RPO, FO and SO diets, respectively. 

Body conformation measurements including body length (BL), chest girth (CG) and withers height (WH) were performed weekly using a measuring tape and following the description by Holman, et al. [28]. Body condition scores (BCS) were subjectively estimated at weekly intervals by the same researcher on a scale of 1 to 5. BCS measurement was as described in detail by Kenyon et al. [29]. Body conformation and BCS measurements were taken while lambs were restrained in a relaxed state with heads comfortably erect and standing stably upon all four legs on flat ground to minimise stress. Individual animal liveweight (LWT) was measured weekly after BCS estimation employing a calibrated Ruddweigh 3000XT Walkover weighing electronic scale with animals standing in a relaxed position.

### 2.4. Feed Analysis

Feed samples from the supplements and lucerne hay were dried, ground and analysed as per standard laboratory methods of AOAC [30] for DM, ash, neutral (NDF) and acid (ADF) detergent fibres, ether extract (EE) and crude protein (CP). Total digestible nutrients (TDN) and conversion of TDN to digestible (DE) and metabolisable (ME) energies were computed as per Bath and Marble [31] and Robinson et al. [32].

### 2.5. Slaughter Protocol and Carcass Characteristic Measurements

All animals were fasted overnight before transporting them to a near-by commercial abattoir (Tasmanian Quality Meats, Cressy, Tasmania, Australia) adjacent to the experimental site in strict compliance with the slaughtering procedures prescribed by the Meat Standards of Australia guidelines. Hot carcasses weights (HCW) were determined immediately after slaughter and the removal of non-edible carcass components (head, hide, intestinal tract, and internal organs). Dressing percentage (DP) was calculated as: DP (%) = (HCW/LWT) × 100. Thereafter, the carcasses were chilled for 24 h at 4 °C and transported to Robinson Meats, Glenorchy, Hobart, Tasmania, Australia for commercial boning out into retail cuts and carcass measurements. Carcass characteristic determination of fat thickness, body wall thickness and rib eye area were taken in accordance with the detailed description by Flakemore et al. [19]. Percentage of boneless, closely trimmed retail cuts (BCTRC) was computed using the equation: %BCTRC = (49.936 − (0.0848 × 2.205 × HCW) − (4.376 × 0.3937 × FD) − (3.53 × 0.3937 × BWT) + (2.456 × 0.155 × REA) where HCW: hot carcass weight; FD: fat thickness; BWT: body wall thickness and REA: rib eye area [33]. Over-the-hooks (OTH) trade value in Australian dollars was computed as HCW × 520 ¢/kg divided by 100 ¢ to produce an average total dollar value per carcass for animals from each treatment group. Five hundred and twenty Australian cents per kilogram (520 ¢/kg) was the amount received for the sale of the lambs used in this study, and is within the range for OTH prices for 2016 [34].

### 2.6. Statistical Analysis

All collected data were analysed using the General Linear Model procedure (PROC GLM) of Statistical Analysis System [35]. The fixed effects of treatment and gender and their second order interactions on growth, body conformation and carcass traits were tested. The initial full statistical model used for the analysis was:

Y = μ + O_i_ + G_j_ + (OG)_ij_ + (OG)^2^_ij_ + (OG)^3^_ij_ + e_ijk_

where

Y = dependent variable,
μ = overall mean,
O_i_ = oil supplementation treatment,
G_k_ = gender, brackets and superscripts represent linear and cubic second-order interactions and e_ijk_ = residual error.

Linear and cubic orthogonal contrasts indicated that gender was not a significant factor, hence it was removed from the final model that assessed the impact of treatment only. Duncan’s multiple range tests were used to determine the differences amongst treatments at a minimum threshold of *p* < 0.05 level. 

## 3. Results

### 3.1. Chemical Composition of Experimental and Basal Feed

The chemical composition of experimental diets is presented in Table 1. DM of all supplemented pellets and basal feed were similar, ranging between 86.8% and 91%. Crude protein content of the different supplemented pellets ranged between 13.5% and 15.6%, which was lower than that in the basal feed (17.1%). ADF and NDF contents of the different supplemented pellets ranged from 7.5% to 10.4% and from 19.0% to 22.2%, respectively, while ADF and NDF content of the basal feed were 36.9% and 47.2%, respectively. In terms of EE content, the level in the supplemented pellets fluctuated between 5.1% and 5.6%, which was at least three-fold higher than the amount in the basal feed (1.5%). ME content of all supplemented pellets was approximately 12.2 (MJ/kg), whilst the basal feed contained 9.08 (MJ/kg) ME. 

### 3.2. Liveweight, Average Daily Gain, Feed Intake and Feed Cost

Liveweight, average daily gain, feed intake responses of prime lambs and feed costs, associated with the different PUFA enriched pellets are shown in Table 2. Liveweight of the animals in all treatments at the beginning of the experiment were similar. However, at the end of the experiment, all animals in the supplemented groups had a higher liveweight than their counterparts in the control group. Furthermore, there was no difference in liveweight among the supplemented groups. The ADG of the control group was only half of that recorded for the supplemented groups. ADG of the SO treatment had the lowest value among the supplemented treatments (190.3 g/head/day). The ratio of hay to concentrate feed intake in lambs supplemented with PUFA enriched pellets was approximately 50:50. The total feed intake of the supplemented animals was 1.70 (kg/head/day), which was significantly higher than that of the control group (1.38 kg/head/day). Lucerne hay conversion efficiency (LCE) of all lambs in the supplemented groups was 4.20 (kg lucerne hay/kg LWT gain per animal), which was only approximately one fourth of the LCE in the control group (16.6 kg lucerne hay/kg LWT gain per animal). There was a significant difference among the supplemented treatments in terms of concentrate feed conversion efficiency (FCE) in which the RBO and SO groups had a higher mean FCE compared to the remaining supplemented treatments. The result also showed that the RBO and CO treatments had the lowest feed cost per unit gain (FCPUG) of AU$3.0/kg, which was only about one third of the FCPUG in the control group and one half of the FCPUG in the SO group. RPO and FO treatments had medium FCPUG of AU$4.1/kg and AU$4.2/kg, respectively.

### 3.3. Body Conformation Traits

All body conformation traits and BCS in all experimental animals did not differ at the beginning of the experiment as depicted in Table 3. However, at the end of the experiment, all animals in the supplemented treatments had significantly greater changes in all body conformation traits and BCS than animals in the control group. There was no difference in any of the changes in body conformation traits and BCS among the supplemented treatments throughout the whole experimental period.

### 3.4. Carcass Characteristics

Carcass characteristics of experimental lambs are demonstrated in Table 4. All the parameters related to carcass characteristics of the supplemented treatments were greater than that of the control group, with the exception of BCTRC% which was greater in the control animals than in the other treatments. There was no difference in pre-slaughter weight, HCW, body wall thickness, rib eye area, BCTRC%, GR fat score and OTH trade among supplemented treatments. The mean dressing percentage significantly differed among supplemented treatments, with the highest value found in the RBO (50.9%) group. Similarly, the mean fat thickness also significantly differed among the supplemented treatments, with the highest value recorded in the lambs supplemented with RPO (6.4 mm) and the lowest value in those supplemented with SO (5.2 mm).

## 4. Discussion

### 4.1. Chemical Composition of Experimental and Basal Feed

The ME content of the basal feed was 9.08 MJ/kg which is lower than the 12.0 MJ/kg proposed for the ideal growth rate [36]. Therefore, concentrate supplementation is needed to maximise lamb growth potential. The CP, ME, NDF and EE content of all supplemented pellets were similar, therefore any differences in lamb growth indicators could be attributed to the different oil sources. The CP content (from 14.0% to 17.1%) in both basal feed and supplemented pellets were well above the 10.7%CP requirement for maintenance and growth [36]. The high CP content in pellets and the increased NDF levels (47.2%) in basal feed provide good fibre and nitrogen sources for rumen microbial growth and contribute to the high growth performance of lambs.

### 4.2. Liveweight, Average Daily Gain, Feed Intake and Feed Cost

Supplementation of prime lambs with PUFA enriched pellets significantly increased LWT and doubled the ADG in the supplemented groups compared to the control group at the end of the feeding trial. Although better protein nutrition can improve lamb performance, we argue that this was not the case in our study. The reason for this is that the supplements were deliberately formulated to have a very similar protein content of approximately 15% and a similar metabolisable energy content of approximately 12 MJ/kg to provide a level playing field for all the supplements. In other words, the supplements were isonitrogenous (similar protein content) and isocaloric (similar energy content). Therefore, this eliminates or minimises any potential differences due to protein or energy. This significant increase in LWT and ADG over and above those in the control group could be explained by the input of supplemented nutrients contained in the pellets, especially ME, minerals and vitamins which better matched the nutrient requirements for growing lambs and increased the total DM and ME intake. This result was in line with the observations in fat-tailed lambs and goats [37,38], which showed that an increase in the level of concentrate resulted in improved ADG. Although there was no significant difference in final LWT between the supplemented treatments, the ADG of the SO treatment was the lowest among the supplemented treatments. Peng et al. [39] investigated the use of different oilseed supplements (sunflower seed, safflower seed, rapeseed, and linseed) in adult ewes and their findings closely agree with the present study in which animals in safflower supplementation group had the lowest ADG in comparison with other treatments. The low ADG and high lucerne intake resulted in the markedly high value of LCE (16.6) in the control group. The poor feed conversion also led to the high FCPUG (AU$9.5/kg) in the control group. The lower ADG in SO treatment resulted in higher FCE value in comparison with the remaining supplemented treatments. The total of LCE and FCE in all the supplemented treatments ranged from 7.44 to 9.3, which is similar to the results of Papi et al. [37], in which lambs had a feed conversion ratio in the range of 7.35 and 9.53. The lowest FCPUG in the RBO and CO treatments can be explained by the lower price of the oils used in preparation of the pellets. Safflower oil was the most expensive ingredient (AU$0.778/kg) which resulted in a higher feed expense in the SO treatment. Similarly, the groups of RPO and FO, with prices of 0.534 and 0.486 AU$/kg, respectively, were in a medium level of feed costs. Feed cost may be an important factor that could influence lamb producers in their decision to supplement lambs with a new feed source or not. However, feed cost depends on many factors such as price and availability of the different feed sources, as well as the supplementation proportion and quality. This study showed that RBO and CO could be used as alternative supplements for lambs, with low feed cost occurring, whilst maintaining comparable lamb ADG value to other treatments.

### 4.3. Body Conformation Traits

The magnitude of changes in body conformation and body condition score parameters in supplemented lambs were higher than those in the control group. As explained previously, the result is likely due to the intake of feed with higher nutrient content by lambs fed diets supplemented with concentrates. The observation herein of no differences occurring in any body conformation traits and body condition score changes between the supplemented treatments, closely aligns with the results of Nguyen et al. [27] who reported that the inclusion of different levels of canola and flaxseed oils in prime lamb diets did not cause any significant differences in body conformation and body condition scores.

### 4.4. Carcass Characteristics

The lower values associated with carcass characteristics of the control lambs could be due to the unbalanced diet and low ME content. Although lucerne hay fed to the lambs in this trial had high CP content (17.1%), it had a relatively lower ME (9.08 MJ/kg) than the ME of 12 MJ/kg required for optimal lamb growth [36]. All the other supplemented treatments had a higher ME of approximately 12.2 MJ/kg which is above the ME requirement for growth and also contained a better roughage-concentrate ratio (approximately 50:50). This trend was observed in the study on different hay-to-concentrate ratios in which the results showed that hot carcass weight, pre-slaughter weight, dressing percentage, and backfat thickness increased when the proportion of concentrates in the ration increased from 30% to 70% [37]. The findings in the present study of no differences in carcass characteristics and OTH among the supplemented treatments agree with the results of Flakemore et al. [19] that examined different levels of oil-infused rice bran supplement in their feeding trial. Those authors also did not find any differences between treatments in terms of carcass characteristics and OTH in the supplemented lambs. Manso et al. [40] also showed that the inclusion of 4% hydrogenated palm oil or sunflower oil in the concentrate did not affect any of the carcass characteristics of lambs. In summary, with the exception of a higher dressing percentage and fat thickness in the RBO and RPO treatments, respectively, the inclusion of different oils in feed pellets did not change the carcass characteristics and OTH of lamb meat.

## 5. Conclusions

Increases in total feed intake, final LWT, ADG, body conformation traits and carcass characteristics were found in prime lambs fed a lucerne-basal and wheat-based pelleted diet enriched with PUFA-containing oil compared to lambs that were fed lucerne alone. Therefore, supplementation of lucerne fed lambs with PUFA enriched pellets is recommended to increase animal production. The use of RBO and CO in fattening prime lambs had comparatively lower feeding costs and did not affect live animal performance, carcass characteristics and OTH trade income in comparison to other sources of PUFA.

## Figures and Tables

**Table 1:** Proximate analysis of the experimental and basal diets.

Chemical Composition (% DM)	Control Lucerne Hay	RBO	CO	RPO	FO	SO
DM	86.8	89.9	91.0	89.7	90.7	89.9
CP	17.1	14.8	14.0	15.6	14.6	14.5
ADF	36.9	7.5	9.4	8.2	10.4	10.0
NDF	47.2	19.0	19.1	20.4	22.2	21.1
EE	1.5	5.5	5.6	5.1	5.6	5.5
ASH	8.4	6.7	6.2	6.5	8.2	8.2
%TDN	60.2	83.1	81.6	82.5	80.8	81.1
DE (Mcal/kg)	2.65	3.65	3.59	3.63	3.56	3.57
ME (MJ/kg)	9.08	12.54	12.32	12.46	12.20	12.25

DM: dry matter; NDF: neutral detergent fibre; ADF: acid detergent fibre; EE: ether extract; CP: crude protein; %TDN: total digestible nutrients; ME: metabolisable energy; RBO, CO, RPO, FO and SO was wheat-based pellet infused with 50 mL/kg DM of oil from rice bran, canola, rumen-protected, flaxseed and safflower sources, respectively. Total digestible nutrients (%TDN) were calculated as TDN (% of DM) = 82.38 − (0.7515 × ADF [% of DM]). Metabolisable energy (ME) was calculated by converting %TDN to digestible energy (DE [Mcal/kg] = %TDN × 0.01 × 4.4) which was converted as ME = (DE (Mcal/kg) × 0.82) × 4.185.

**Table 2:** Liveweight, average daily gain, feed intake and feed costs per unit gain of prime lambs fed various PUFA enriched pellets *.

Parameters	Control	RBO	CO	RPO	FO	SO	SEM
Initial LWT (kg)	37.6	38.6	37.6	38.3	38.3	38.6	0.59
Final LWT (kg)	42.4 ^b^	48.9 ^a^	48.9 ^a^	50.3 ^a^	49.8 ^a^	48.2 ^a^	0.79
ADG (g)	94.3 ^c^	205.7 ^ab^	226.3 ^a^	240.0 ^a^	230.2 ^a^	190.3 ^b^	11.22
Lucerne hay intake (kg DM/head/day)	1.38 ^a^	0.79 ^b^	0.86 ^b^	0.88 ^b^	0.95 ^b^	0.85 ^b^	0.08
Concentrate intake (kg DM/head/day)	-	0.86	0.82	0.83	0.84	0.86	0.05
Total intake (kg DM/head/day)	1.38 ^b^	1.64 ^a^	1.68 ^a^	1.71 ^a^	1.79 ^a^	1.71 ^a^	0.07
LCE	16.6 ^a^	4.0 ^b^	3.9 ^b^	3.8 ^b^	4.2 ^b^	4.6 ^b^	0.79
FCE	-	4.3 ^ab^	3.7 ^b^	3.6 ^b^	3.7 ^b^	4.7 ^a^	0.24
FCPUG (AU$/kg)	9.5 ^a^	3.0 ^d^	3.0 ^d^	4.1 ^c^	4.2 ^c^	6.3 ^b^	0.23

LWT: liveweight; ADG: average daily gain; LCE: lucerne hay conversion efficiency (kg DM lucerne hay/kg gain per animal); FCE: concentrate feed conversion efficiency (kg DM concentrate/kg gain per animal); FCPUG: feed cost per unit gain (concentrate and lucerne hay cost of feed/kg; LWT: gain (AU$/kg) per animal); SEM: standard error of the means. All other abbreviations as explained in Table 1. * Values within the same row not bearing a common superscript differ (*p* < 0.05).

**Table 3:** Changes in body conformation traits of prime lambs fed various PUFA enriched pellets *.

Body Conformation Traits	Control	RBO	CO	RPO	FO	SO	SEM
Initial CG (cm)	77.3	79.0	77.3	78.8	78.4	78.6	0.63
ΔCG (cm)	4.9 ^b^	8.6 ^a^	9.3 ^a^	8.8 ^a^	9.1 ^a^	8.5 ^a^	0.63
Initial WH (cm)	61.6	60.9	61.2	61.4	62.0	61.1	0.44
ΔWH (cm)	3.9 ^b^	5.2 ^a^	5.6 ^a^	5.6 ^a^	5.5 ^a^	5.2 ^a^	0.35
Initial BL (cm)	61.8	62.7	62.1	62.7	62.9	62.3	0.39
ΔBL (cm)	4.1 ^b^	5.2 ^a^	5.1 ^a^	5.2 ^a^	5.4 ^a^	5.5 ^a^	0.33
Initial BCS	2.63	2.67	2.58	2.63	2.63	2.67	0.07
ΔBCS	−0.21 ^b^	0.96 ^a^	1.00 ^a^	1.13 ^a^	1.21 ^a^	1.08 ^a^	0.13

Δ: change in; CG: chest girth; WH: withers height; BL: body length; BCS: body condition score. All other abbreviations as in Table 1 and Table 2. * Values within the same row bearing different superscripts differ (*p* < 0.05).

**Table 4:** Carcass characteristics of prime lambs fed various PUFA enriched pellets *.

Items	Control	RBO	CO	RPO	FO	SO	SEM
Pre-slaughter weight (kg)	40.4 ^b^	47.0 ^a^	46.1 ^a^	47.6 ^a^	47.7 ^a^	47.3 ^a^	0.72
HCW (kg)	19.4 ^b^	24.9 ^a^	23.7 ^a^	24.6 ^a^	24.5 ^a^	23.9 ^a^	0.47
Dressing percentage (%)	45.7 ^c^	50.9 ^a^	48.4 ^b^	48.9 ^b^	49.1 ^b^	49.5 ^ab^	0.53
Fat thickness (mm)	4.0 ^c^	5.7 ^ab^	5.5 ^ab^	6.4 ^a^	6.2 ^ab^	5.2 ^b^	0.38
Body wall thickness (mm)	16.4 ^b^	21.8 ^a^	21.8 ^a^	22.8 ^a^	20.4 ^a^	21.7 ^a^	1.03
Rib eye area (cm2)	14.8 ^b^	17.0 ^a^	15.8 ^ab^	16.4 ^a^	16.1 ^ab^	16.1 ^ab^	0.44
BCTRC%	49.0 ^a^	47.7 ^b^	47.5 ^b^	47.3 ^b^	47.6 ^b^	47.7 ^b^	0.27
GR fat score (1–5)	2.5 ^b^	3.7 ^a^	3.4 ^a^	3.7 ^a^	3.5 ^a^	3.3 ^a^	0.17
OTH trade (AU$)	100.6 ^b^	129.3 ^a^	123.1 ^a^	127.8 ^a^	127.2 ^a^	124 ^a^	2.43

Pre-slaughter weight: the weight of animals prior to transport for slaughter; HCW: hot carcass weight; BCTRC%: boneless, closely trimmed retail cuts; OTH: over the hooks trade (this was based on 520 AU¢ return per kg of HCW). All other abbreviations as in Table 1 and Table 2. * Values within the same row bearing different superscripts differ (*p* < 0.05).
